# Supplementary material for: The Ruminal Microbiome Alterations Associated with Diet-Induced Milk Fat Depression and Milk Fat Globule Size Reduction in Dairy Goats
Source: Animals (Basel). 2024 Sep 9;14(17):2614. doi: 10.3390/ani14172614 (PMC11393860; doi:10.3390/ani14172614)
Supplement: Supplementary file 1 [file animals-14-02614-s001.zip › animals-3155582-supplementary.pdf]

Table S1. Ingredients and chemical composition of the basal diets of dairy goats

Composition	Content %
Ingredient	
Corn silage	17.00
Alfalfa hay	23.00
Peanut straw	25.00
Corn	8.30
Wheat bran	10.16
Soybean meal	14.74
$NaHCO_3$	0.50
$CaHCO_3$	0.21
Limestone	0.09
Premix [a]	1.00
Chemical composition	
Lactation net energy, (MJ/kg)	5.97
Crude protein	16.50
Neutral detergent fiber	44.52
Acid detergent fiber	27.58
Ca	0.61
P	0.45

[a] Composition per kg diet: 50,000 IU of vitamin A; 25 mg of vitamin B_1; 95 mg of vitamin B_2; 40 mg of vitamin B_5; 25 mg of vitamin B_6; 0.38 mg of vitamin B_{12}; 17,500 IU of vitamin D_3; 100 IU of vitamin E_1; 12.5 mg of vitamin K_3; 375 mg of Cu; 1,750 mg of Fe; 625 mg of Mn; 2,000 mg of Zn; 3.5 mg of I; 3.75 mg of Se.

Nutrient levels are based on dry matter, crude protein, crude fat, neutral detergent fiber, acidic detergent fiber, etc. are all measured values, and lactation net energy is calculated value.

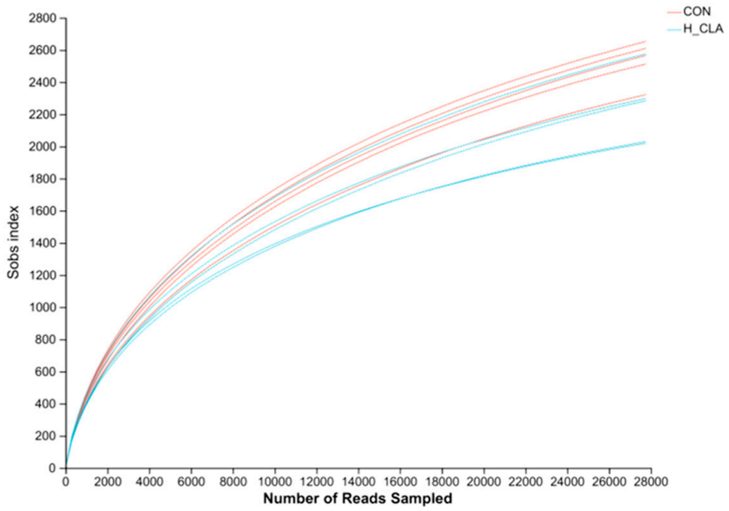

Figure S1 Number of tags sampled

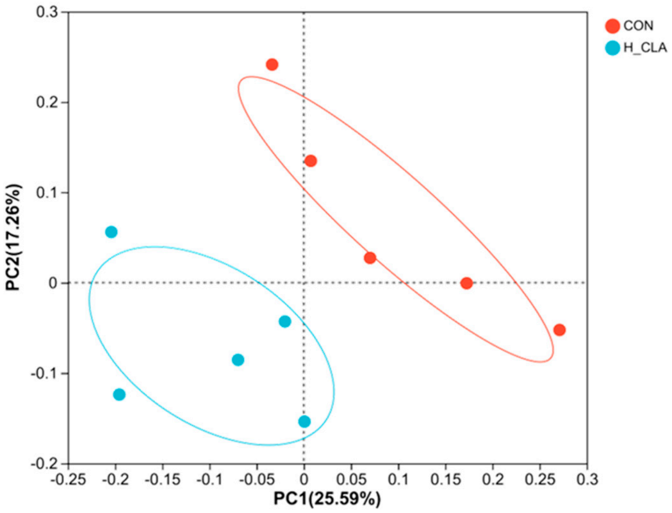

Figure S2 Principal coordinate analysis (PCoA). The unweighted UniFrac distance metrics analysis between the rumen bacteria in CON and H-CLA group
